# The genome sequence of a muscid fly, *Hydrotaea cyrtoneurina *(Zetterstedt, 1845)

**DOI:** 10.12688/wellcomeopenres.20638.1

**Published:** 2024-02-19

**Authors:** Steven Falk, Andrzej Grzywacz, University of Oxford and Wytham Woods Genome Acquisition Lab, Darwin Tree of Life Barcoding collective, Wellcome Sanger Institute Tree of Life Management, Samples and Laboratory team, Wellcome Sanger Institute Scientific Operations: Sequencing Operations, Wellcome Sanger Institute Tree of Life Core Informatics team, Tree of Life Core Informatics collective, Darwin Tree of Life Consortium

**Affiliations:** 1 Independent researcher, Wallingford, England, UK; 2 Nicolaus Copernicus University in Toruń, Toruń, Poland

**Keywords:** Hydrotaea cyrtoneurina, muscid fly, genome sequence, chromosomal, Diptera

## Abstract

We present a genome assembly from an individual female *Hydrotaea cyrtoneurina* (muscid fly; Arthropoda; Insecta; Diptera; Muscidae). The genome sequence is 575.2 megabases in span. Most of the assembly is scaffolded into 6 chromosomal pseudomolecules. The mitochondrial genome has also been assembled and is 19.6 kilobases in length.

## Species taxonomy

Eukaryota; Opisthokonta; Metazoa; Eumetazoa; Bilateria; Protostomia; Ecdysozoa; Panarthropoda; Arthropoda; Mandibulata; Pancrustacea; Hexapoda; Insecta; Dicondylia; Pterygota; Neoptera; Endopterygota; Diptera; Brachycera; Muscomorpha; Eremoneura; Cyclorrhapha; Schizophora; Calyptratae; Muscoidea; Muscidae; Azeliinae; Azeliini; Hydrotaea; *Hydrotaea cyrtoneurina* (Zetterstedt, 1845) (NCBI:txid559647).

## Background

*Hydrotaea cyrtoneurina* (Zetterstedt, 1845) is a representative of the muscid genus *Hydrotaea* Robineau-Desvoidy, 1830 (Diptera: Muscidae). The majority of males of *Hydrotaea* are known from a peculiar foreleg modification, potentially used for grasping females during mating ( Gregor *et al.*, 2002). Larvae are facultative carnivores feeding on decomposing organic tissues or preying on concomitant species. However, some species are obligatory predators with reduced number of free-living larval instars ( Skidmore, 1985).

*Hydrotaea cyrtoneurina* distribution is restricted to the Palaearctic region, where it is widespread throughout Europe and eastwards to Siberia, India and the Korean Peninsula ( Pont, 1986; Sorokina & Pont, 2010). It is a frequently occurring insect throughout the Archipelago of Britain and Ireland, with flight period mostly from April to October ( d’Assis Fonseca, 1968). Adults are closely associated with badger setts, have been reported from fox faeces ( Gregor *et al.*, 2002), and are attracted to carrion and decomposing animal tissues ( Grzywacz *et al.*, 2017). During carrion succession experiments, it has been recognised significantly associated with bloated stage of decomposition ( Matuszewski *et al.*, 2010). The species lacks breeding records from animal carrion and human cadavers ( Grzywacz *et al.*, 2017; Matuszewski *et al.*, 2008), yet larvae, potentially facultative predators with three fee-living larval stages, successfully develop on decomposing animal tissues ( Grzywacz, 2013).

The genome presented here will serve as a source of information for phylogenomic purposes and may be used in comparative genomic studies to answer questions regarding biological adaptations in *Hydrotaea* species.

We present a chromosomally complete genome sequence for *Hydrotaea cyrtoneurina*, based on one female specimen from Wytham Woods, as part of the Darwin Tree of Life Project. This project is a collaborative effort to sequence all named eukaryotic species in the Atlantic Archipelago of Britain and Ireland.

## Genome sequence report

**Figure 1.**
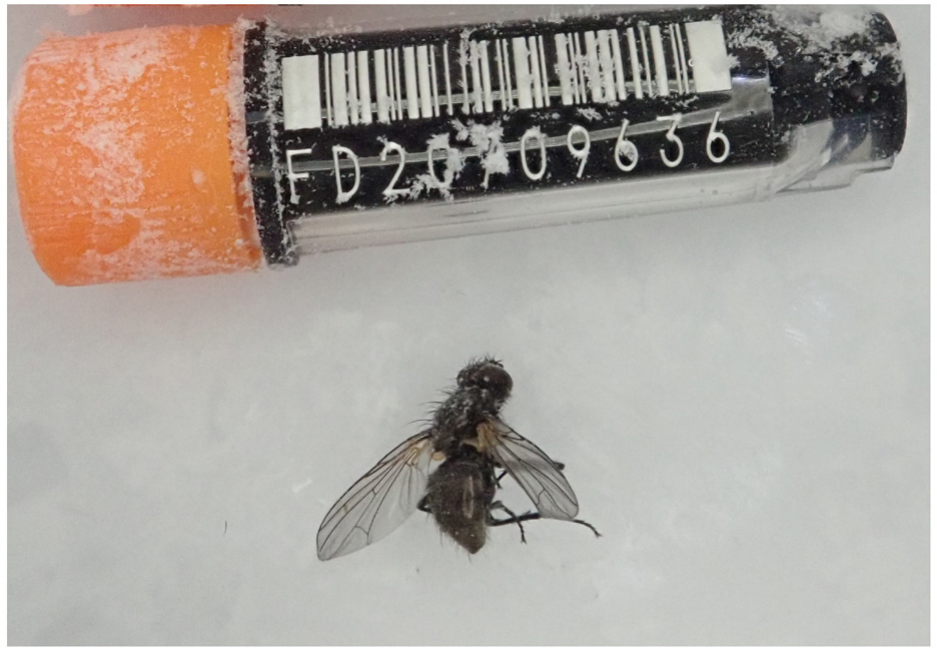
Photograph of the *Hydrotaea cyrtoneurina* (idHydCyrt1) specimen used for genome sequencing

The genome was sequenced from one female *Hydrotaea cyrtoneurina* ( Figure 1) collected from Wytham Woods, Oxfordshire, UK (51.77, –1.31). A total of 30-fold coverage in Pacific Biosciences single-molecule HiFi long reads was generated. Primary assembly contigs were scaffolded with chromosome conformation Hi-C data. Manual assembly curation corrected 37 missing joins or mis-joins and removed 3 haplotypic duplications, reducing the scaffold number by 45.24%.

**Figure 2.**
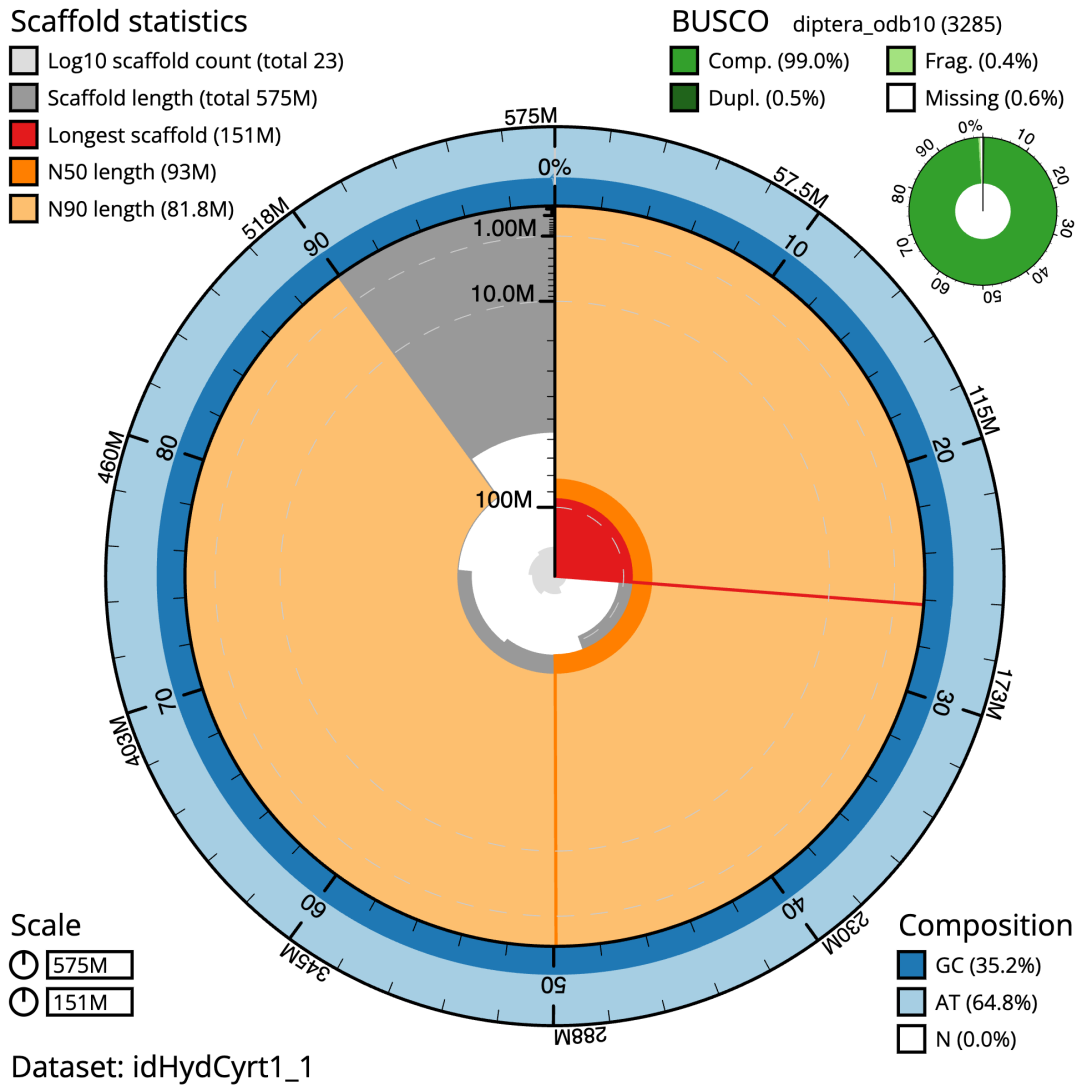
Genome assembly of *Hydrotaea cyrtoneurina*, idHydCyrt1.1: metrics. The BlobToolKit Snailplot shows N50 metrics and BUSCO gene completeness. The main plot is divided into 1,000 size-ordered bins around the circumference with each bin representing 0.1% of the 575,214,542 bp assembly. The distribution of scaffold lengths is shown in dark grey with the plot radius scaled to the longest scaffold present in the assembly (150,892,273 bp, shown in red). Orange and pale-orange arcs show the N50 and N90 scaffold lengths (92,963,629 and 81,795,257 bp), respectively. The pale grey spiral shows the cumulative scaffold count on a log scale with white scale lines showing successive orders of magnitude. The blue and pale-blue area around the outside of the plot shows the distribution of GC, AT and N percentages in the same bins as the inner plot. A summary of complete, fragmented, duplicated and missing BUSCO genes in the diptera_odb10 set is shown in the top right. An interactive version of this figure is available at https://blobtoolkit.genomehubs.org/view/idHydCyrt1_1/dataset/idHydCyrt1_1/snail.

**Figure 3.**
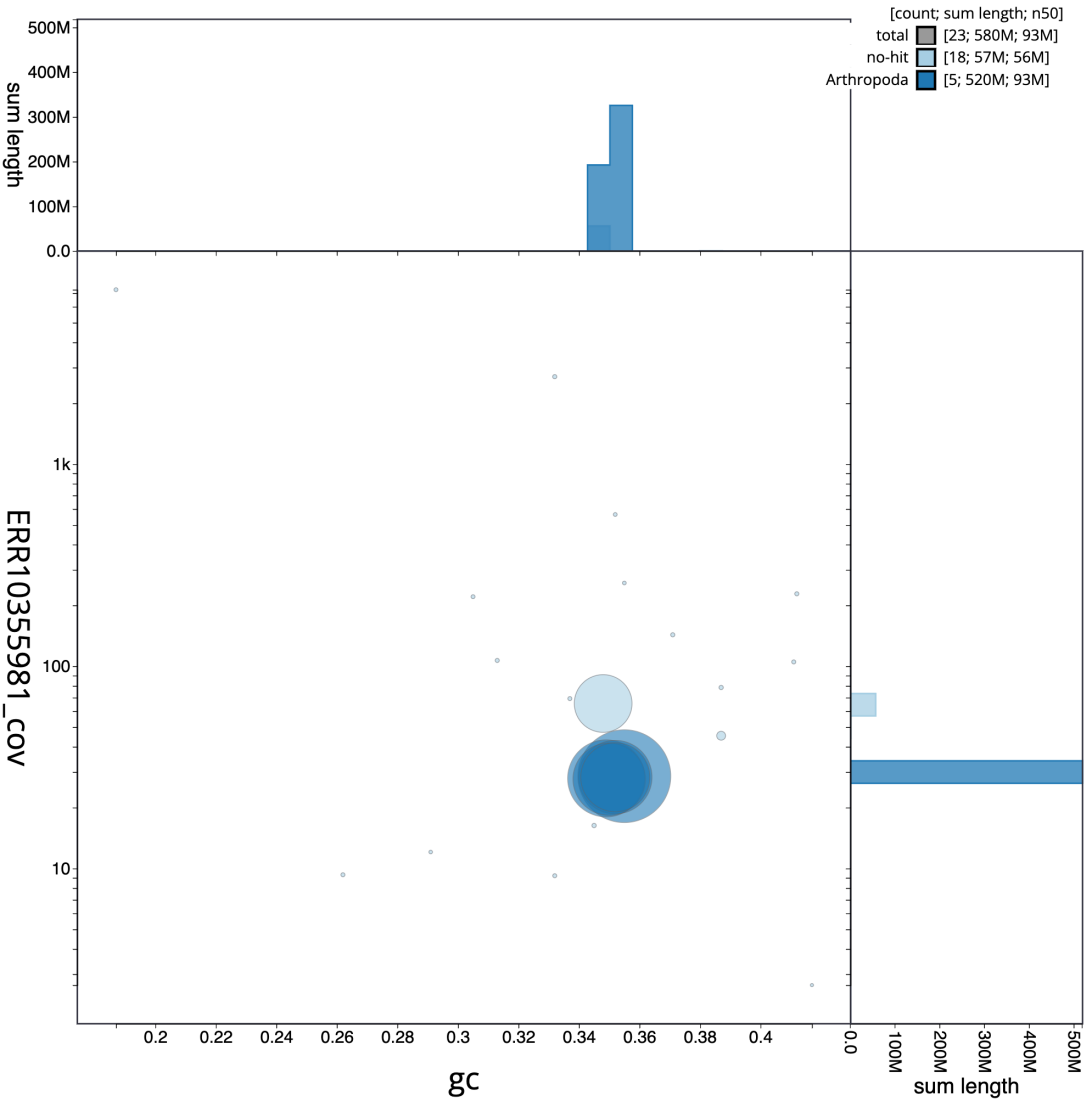
Genome assembly of *Hydrotaea cyrtoneurina*, idHydCyrt1.1: BlobToolKit GC-coverage plot. Scaffolds are coloured by phylum. Circles are sized in proportion to scaffold length. Histograms show the distribution of scaffold length sum along each axis. An interactive version of this figure is available at https://blobtoolkit.genomehubs.org/view/idHydCyrt1_1/dataset/idHydCyrt1_1/blob.

**Figure 4.**
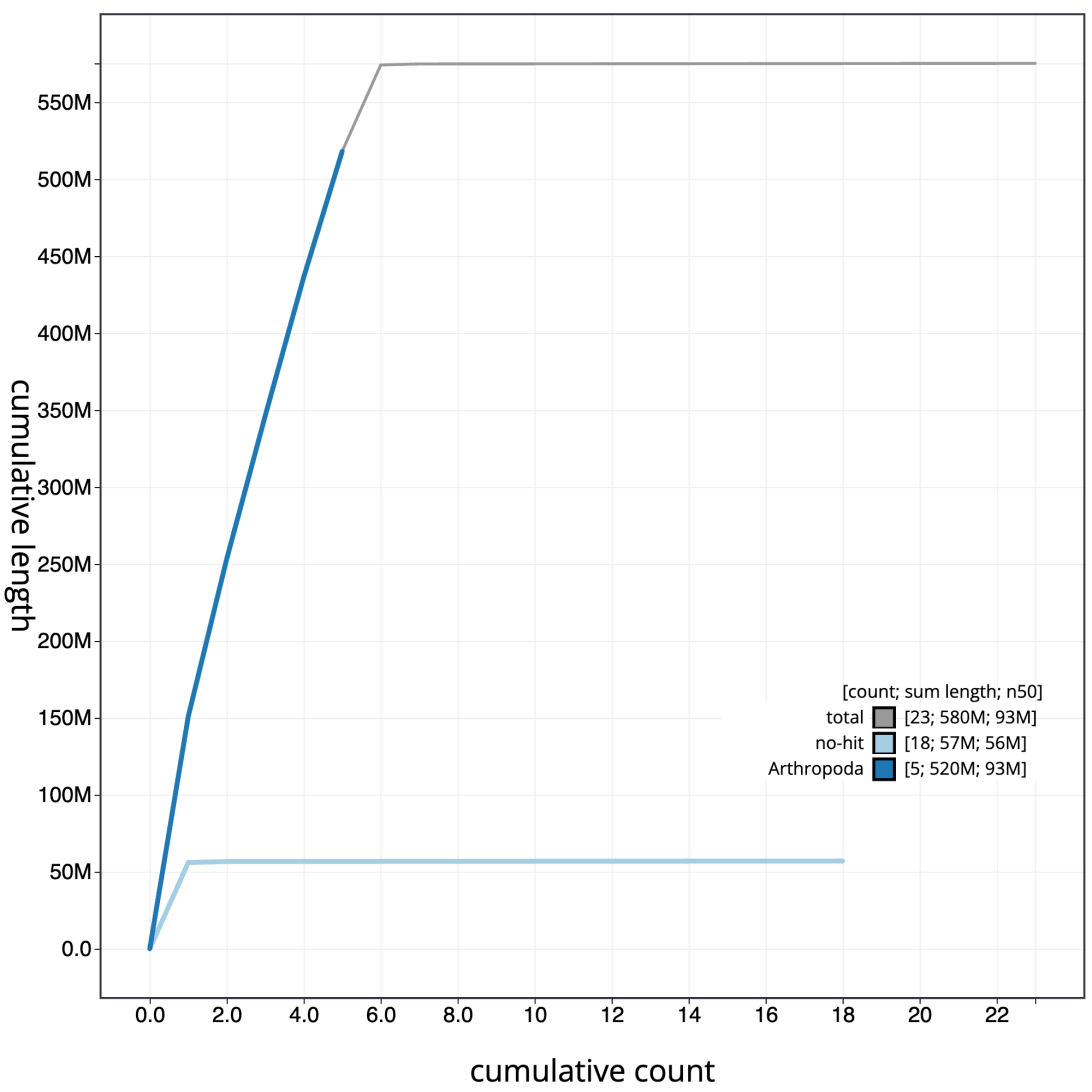
Genome assembly of *Hydrotaea cyrtoneurina*, idHydCyrt1.1: BlobToolKit cumulative sequence plot. The grey line shows cumulative length for all scaffolds. Coloured lines show cumulative lengths of scaffolds assigned to each phylum using the buscogenes taxrule. An interactive version of this figure is available at https://blobtoolkit.genomehubs.org/view/idHydCyrt1_1/dataset/idHydCyrt1_1/cumulative.

**Figure 5.**
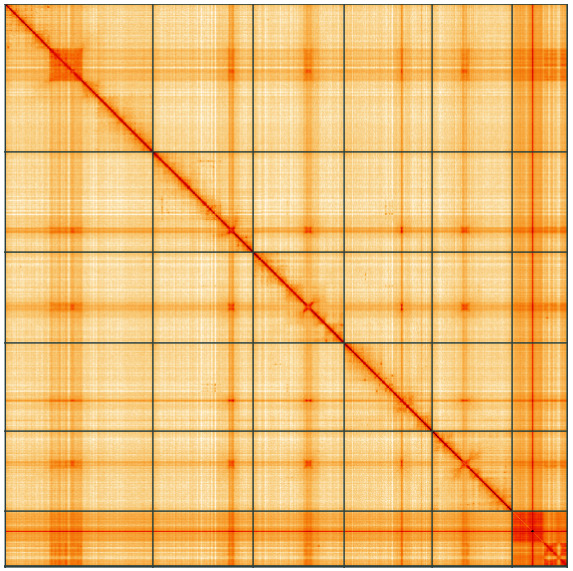
Genome assembly of *Hydrotaea cyrtoneurina*, idHydCyrt1.1: Hi-C contact map of the idHydCyrt1.1 assembly, visualised using HiGlass. Chromosomes are shown in order of size from left to right and top to bottom. An interactive version of this figure may be viewed at https://genome-note-higlass.tol.sanger.ac.uk/l/?d=C1KA3sKITRG3i66TjhBUaQ.

The final assembly has a total length of 575.2 Mb in 22 sequence scaffolds with a scaffold N50 of 93.0 Mb ( Table 1). The snailplot in Figure 2 provides a summary of the assembly statistics, while the distribution of assembly scaffolds on GC proportion and coverage is shown in Figure 3. The cumulative assembly plot in Figure 4 shows curves for subsets of scaffolds assigned to different phyla. Most (99.93%) of the assembly sequence was assigned to 6 chromosomal-level scaffolds, representing 6 autosomes. Chromosome-scale scaffolds confirmed by the Hi-C data are named in order of size ( Figure 5; Table 2). While not fully phased, the assembly deposited is of one haplotype. Contigs corresponding to the second haplotype have also been deposited. The mitochondrial genome was also assembled and can be found as a contig within the multifasta file of the genome submission.

**Table 1.** Genome data for *Hydrotaea cyrtoneurina*, idHydCyrt1.1

Project accession data		
Assembly identifier	idHydCyrt1.1	
Species	*Hydrotaea cyrtoneurina*	
Specimen	idHydCyrt1	
NCBI taxonomy ID	559647	
BioProject	PRJEB56364	
BioSample ID	SAMEA10157814	
Isolate information	idHydCyrt1, female: thorax (DNA sequencing), head (Hi-C sequencing), abdomen (RNA sequencing)	
Assembly metrics *		*Benchmark*
Consensus quality (QV)	61.3	*≥ 50*
*k*-mer completeness	100.0%	*≥ 95%*
BUSCO **	C:99.0%[S:98.6%,D:0.5%], F:0.4%,M:0.6%,n:3,285	*C ≥ 95%*
Percentage of assembly mapped to chromosomes	99.93%	*≥ 95%*
Sex chromosomes	Not identified	*localised* *homologous pairs*
Organelles	Mitochondrial genome: 19.6 kb	*complete single* *alleles*
Raw data accessions		
PacificBiosciences SEQUEL II	ERR10355981	
Hi-C Illumina	ERR10313049	
PolyA RNA-Seq Illumina	ERR10378037	
Genome assembly		
Assembly accession	GCA_958296145.1	
*Accession of alternate* *haplotype*	GCA_958295795.1	
Span (Mb)	575.2	
Number of contigs	149	
Contig N50 length (Mb)	8.1	
Number of scaffolds	22	
Scaffold N50 length (Mb)	93.0	
Longest scaffold (Mb)	151.56	

* Assembly metric benchmarks are adapted from column VGP-2020 of “Table 1: Proposed standards and metrics for defining genome assembly quality” from ( Rhie *et al.*, 2021). ** BUSCO scores based on the diptera_odb10 BUSCO set using version 5.3.2. C = complete [S = single copy, D = duplicated], F = fragmented, M = missing, n = number of orthologues in comparison. A full set of BUSCO scores is available at https://blobtoolkit.genomehubs.org/view/idHydCyrt1_1/dataset/idHydCyrt1_1/busco.

**Table 2.** Chromosomal pseudomolecules in the genome assembly of *Hydrotaea cyrtoneurina*, idHydCyrt1.

INSDC accession	Chromosome	Length (Mb)	GC%
OY282512.1	1	150.89	35.5
OY282513.1	2	102.45	35.0
OY282514.1	3	92.96	35.0
OY282515.1	4	90.06	35.0
OY282516.1	5	81.8	35.0
OY282517.1	6	56.04	35.0
OY282518.1	MT	0.02	19.0

The estimated Quality Value (QV) of the final assembly is 61.3 with *k*-mer completeness of 100.0%, and the assembly has a BUSCO v5.3.2 completeness of 99.0% (single = 98.6%, duplicated = 0.5%), using the diptera_odb10 reference set ( *n* = 3,285).

Metadata for specimens, barcode results, spectra estimates, sequencing runs, contaminants and pre-curation assembly statistics are given at https://links.tol.sanger.ac.uk/species/559647.

## Methods

### Sample acquisition and nucleic acid extraction

A female *Hydrotaea cyrtoneurina* (specimen ID Ox001224, ToLID idHydCyrt1) was collected from Wytham Woods, Oxfordshire (biological vice-county Berkshire), UK (latitude 51.77, longitude –1.31) on 2021-04-19 using a net. The specimen was collected and identified by Steven Falk (independent researcher) and preserved on dry ice.

The workflow for high molecular weight (HMW) DNA extraction at the Wellcome Sanger Institute (WSI) includes a sequence of core procedures: sample preparation; sample homogenisation, DNA extraction, fragmentation, and clean-up. In sample preparation, the idHydCyrt1 sample was weighed and dissected on dry ice ( Jay *et al.*, 2023). Tissue from the thorax was homogenised using a PowerMasher II tissue disruptor ( Denton *et al.*, 2023a). HMW DNA was extracted using the Automated MagAttract v1 protocol ( Sheerin *et al.*, 2023). DNA was sheared into an average fragment size of 12–20 kb in a Megaruptor 3 system with speed setting 30 ( Todorovic *et al.*, 2023). Sheared DNA was purified by solid-phase reversible immobilisation ( Strickland *et al.*, 2023): in brief, the method employs a 1.8X ratio of AMPure PB beads to sample to eliminate shorter fragments and concentrate the DNA. The concentration of the sheared and purified DNA was assessed using a Nanodrop spectrophotometer and Qubit Fluorometer and Qubit dsDNA High Sensitivity Assay kit. Fragment size distribution was evaluated by running the sample on the FemtoPulse system.

RNA was extracted from abdomen tissue of idHydCyrt1 in the Tree of Life Laboratory at the WSI using the RNA Extraction: Automated MagMax™ *mir*Vana protocol ( do Amaral *et al.*, 2023). The RNA concentration was assessed using a Nanodrop spectrophotometer and a Qubit Fluorometer using the Qubit RNA Broad-Range Assay kit. Analysis of the integrity of the RNA was done using the Agilent RNA 6000 Pico Kit and Eukaryotic Total RNA assay.

Protocols developed by the WSI Tree of Life laboratory are publicly available on protocols.io ( Denton *et al.*, 2023b).

### Sequencing

Pacific Biosciences HiFi circular consensus DNA sequencing libraries were constructed according to the manufacturers’ instructions. Poly(A) RNA-Seq libraries were constructed using the NEB Ultra II RNA Library Prep kit. DNA and RNA sequencing was performed by the Scientific Operations core at the WSI on Pacific Biosciences SEQUEL II (HiFi) and Illumina NovaSeq 6000 (RNA-Seq) instruments. Hi-C data were also generated from head tissue of idHydCyrt1 using the Arima2 kit and sequenced on the Illumina NovaSeq 6000 instrument.

### Genome assembly, curation and evaluation

Assembly was carried out with Hifiasm ( Cheng *et al.*, 2021) and haplotypic duplication was identified and removed with purge_dups ( Guan *et al.*, 2020). The assembly was then scaffolded with Hi-C data ( Rao *et al.*, 2014) using YaHS ( Zhou *et al.*, 2023). The assembly was checked for contamination and corrected using the gEVAL system ( Chow *et al.*, 2016) as described previously ( Howe *et al.*, 2021). Manual curation was performed using gEVAL, HiGlass ( Kerpedjiev *et al.*, 2018) and Pretext ( Harry, 2022). The mitochondrial genome was assembled using MitoHiFi ( Uliano-Silva *et al.*, 2023), which runs MitoFinder ( Allio *et al.*, 2020) or MITOS ( Bernt *et al.*, 2013) and uses these annotations to select the final mitochondrial contig and to ensure the general quality of the sequence.

A Hi-C map for the final assembly was produced using bwa-mem2 ( Vasimuddin *et al.*, 2019) in the Cooler file format ( Abdennur & Mirny, 2020). To assess the assembly metrics, the *k*-mer completeness and QV consensus quality values were calculated in Merqury ( Rhie *et al.*, 2020). This work was done using Nextflow ( Di Tommaso *et al.*, 2017) DSL2 pipelines “sanger-tol/readmapping” ( Surana *et al.*, 2023a) and “sanger-tol/genomenote” ( Surana *et al.*, 2023b). The genome was analysed within the BlobToolKit environment ( Challis *et al.*, 2020) and BUSCO scores ( Manni *et al.*, 2021; Simão *et al.*, 2015) were calculated.

Table 3 contains a list of relevant software tool versions and sources.

**Table 3.** Software tools: versions and sources.

Software tool	Version	Source
BlobToolKit	4.2.1	https://github.com/blobtoolkit/blobtoolkit
BUSCO	5.3.2	https://gitlab.com/ezlab/busco
Hifiasm	0.16.1-r375	https://github.com/chhylp123/hifiasm
HiGlass	1.11.6	https://github.com/higlass/higlass
Merqury	MerquryFK	https://github.com/thegenemyers/MERQURY.FK
MitoHiFi	2	https://github.com/marcelauliano/MitoHiFi
PretextView	0.2	https://github.com/wtsi-hpag/PretextView
purge_dups	1.2.3	https://github.com/dfguan/purge_dups
sanger-tol/genomenote	v1.0	https://github.com/sanger-tol/genomenote
sanger-tol/readmapping	1.1.0	https://github.com/sanger-tol/readmapping/tree/1.1.0
YaHS	yahs-1.1.91eebc2	https://github.com/c-zhou/yahs

### Wellcome Sanger Institute – Legal and Governance

The materials that have contributed to this genome note have been supplied by a Darwin Tree of Life Partner. The submission of materials by a Darwin Tree of Life Partner is subject to the **‘Darwin Tree of Life Project Sampling Code of Practice’**, which can be found in full on the Darwin Tree of Life website here. By agreeing with and signing up to the Sampling Code of Practice, the Darwin Tree of Life Partner agrees they will meet the legal and ethical requirements and standards set out within this document in respect of all samples acquired for, and supplied to, the Darwin Tree of Life Project.

Further, the Wellcome Sanger Institute employs a process whereby due diligence is carried out proportionate to the nature of the materials themselves, and the circumstances under which they have been/are to be collected and provided for use. The purpose of this is to address and mitigate any potential legal and/or ethical implications of receipt and use of the materials as part of the research project, and to ensure that in doing so we align with best practice wherever possible. The overarching areas of consideration are:

•      Ethical review of provenance and sourcing of the material

•      Legality of collection, transfer and use (national and international)

Each transfer of samples is further undertaken according to a Research Collaboration Agreement or Material Transfer Agreement entered into by the Darwin Tree of Life Partner, Genome Research Limited (operating as the Wellcome Sanger Institute), and in some circumstances other Darwin Tree of Life collaborators.

## Data Availability

European Nucleotide Archive: *Hydrotaea cyrtoneurina*. Accession number PRJEB56364; https://identifiers.org/ena.embl/PRJEB56364 ( Wellcome Sanger Institute, 2023). The genome sequence is released openly for reuse. The *Hydrotaea cyrtoneurina* genome sequencing initiative is part of the Darwin Tree of Life (DToL) project. All raw sequence data and the assembly have been deposited in INSDC databases. The genome will be annotated using available RNA-Seq data and presented through the Ensembl pipeline at the European Bioinformatics Institute. Raw data and assembly accession identifiers are reported in Table 1.

## Author information

Members of the University of Oxford and Wytham Woods Genome Acquisition Lab are listed here: https://doi.org/10.5281/zenodo.7125292.

Members of the Darwin Tree of Life Barcoding collective are listed here: https://doi.org/10.5281/zenodo.4893703.

Members of the Wellcome Sanger Institute Tree of Life Management, Samples and Laboratory team are listed here: https://doi.org/10.5281/zenodo.10066175.

Members of Wellcome Sanger Institute Scientific Operations: Sequencing Operations are listed here: https://doi.org/10.5281/zenodo.10043364.

Members of the Wellcome Sanger Institute Tree of Life Core Informatics team are listed here: https://doi.org/10.5281/zenodo.10066637.

Members of the Tree of Life Core Informatics collective are listed here: https://doi.org/10.5281/zenodo.5013541.

Members of the Darwin Tree of Life Consortium are listed here: https://doi.org/10.5281/zenodo.4783558.
