# Supplementary material for: The genome sequence of a muscid fly, Hydrotaea cyrtoneurina (Zetterstedt, 1845)
Source: Wellcome Open Res. 2024 Feb 19;9:60. [Version 1] doi: 10.12688/wellcomeopenres.20638.1 (PMC11079579; doi:10.12688/wellcomeopenres.20638.1)
Supplement: Peer review history

# Reviewer report: Reviewer response for version 1

**DOI:** 10.21956/wellcomeopenres.22841.r79257

**Published:** 2024-05-08

**Authors:** Andrés López Rubio

**Affiliations:** 1 Tecnológico de Antioquia – University Institute, Antioquia, Colombia

This article presents a genome sequence for *Hydrotaea cyrtoneurina, *a muscid fly with paleartic distribution. All the materials and method using the Pacific Biosciences platform were clear, as well as the assembly stratergies.

 For future studies, have you considered the enrichment of this genome by including specimens at different locations along the species distribution.?

Are sufficient details of methods and materials provided to allow replication by others?

Yes

Is the rationale for creating the dataset(s) clearly described?

Yes

Are the datasets clearly presented in a useable and accessible format?

Yes

Are the protocols appropriate and is the work technically sound?

Yes

Reviewer Expertise:

evolution, phylogenetics, molecular phylogenetics, genomics

I confirm that I have read this submission and believe that I have an appropriate level of expertise to confirm that it is of an acceptable scientific standard.

# Reviewer report: Reviewer response for version 1

**DOI:** 10.21956/wellcomeopenres.22841.r79251

**Published:** 2024-04-17

**Authors:** William Reid

**Affiliations:** 1 Biochemistry, SUNY at Buffalo, Buffalo, New York, USA

In the data note "The genome sequence of a muscid fly, Hydrotaea cyrtoneurina (Zetterstedt, 1845)", Falk et al present the genome assembly for Hydrotaea cyrtoneurina using PacBio long read sequencing and HiC scaffolding to reconstruct six autosomes. For their strategy, they divided the animal into the three soma, and used the head, thorax, and abdomen for the starting material for the HiC, genomic, and transcriptomic libraries, respectively. The abdomen would contain much of the metabolically active fat body (as well as other tissues) and be a good choice for the RNA library. Further, the omission of the abdomen from the genomic library avoids problems with the mating status of the female for the genomic assembly. Overall, they followed the standard pipeline for the Tree of Life consortium, which also has all of the protocols submitted to protocols.io. All data links are valid and available.

Are sufficient details of methods and materials provided to allow replication by others?

Yes

Is the rationale for creating the dataset(s) clearly described?

Yes

Are the datasets clearly presented in a useable and accessible format?

Yes

Are the protocols appropriate and is the work technically sound?

Yes

Reviewer Expertise:

Insect transgenics

I confirm that I have read this submission and believe that I have an appropriate level of expertise to confirm that it is of an acceptable scientific standard.
